# Case report: a patient with spontaneous spinal epidural hematoma recovered after conservative treatment

**DOI:** 10.3389/fmed.2025.1509903

**Published:** 2025-02-04

**Authors:** Jingge Cheng, Bo Li, Shuaiye Liu, Yiming Liu, Yipeng Zhao

**Affiliations:** ^1^Department of Emergency and Disaster Medicine, Seventh Affiliated Hospital, Sun Yat-sen University, Shenzhen, China; ^2^Department of Cardiology, Seventh Affiliated Hospital, Sun Yat-sen University, Shenzhen, China; ^3^Department of Pathology, Seventh Affiliated Hospital, Sun Yat-sen University, Shenzhen, China

**Keywords:** case report, spontaneous spinal epidural hematoma, stroke, stroke mimic, conservative treatment

## Abstract

Spontaneous spinal epidural hematoma is a rare condition, akin to a stroke, which can manifest as hemiplegia. It cannot be diagnosed by head CT scan and is prone to misdiagnosis as a cerebral stroke, potentially leading to the activation of thrombolysis and exacerbation of the condition. This paper reports a case of a patient with this disease, a 68 years-old male who presented with sudden neck pain weakness, and numbness in the left limbs for 2 h. The symptoms were similar to those of a stroke. A head CT scan showed no significant abnormalities, but the clinical presentation of neck pain alerted the physician, who did not hastily administer thrombolysis. Instead, they quickly performed further examinations, including neck vessel ultrasound and neck CT/MRI, to rule out a cerebral stroke. The patient was ultimately diagnosed with spontaneous spinal epidural hematoma in the neck. The patient was transferred to the ICU for conservative treatment, and a follow-up neck MRI the next day revealed a significant reduction in the hematoma, with a corresponding improvement in clinical symptoms. This case report supports the diagnostic value of CT scans for spinal epidural hematoma and shares a conservative treatment plan for patients with high spinal cord lesions.

## 1 Introduction

Spontaneous spinal epidural hematomas (SSEH) is a rare disease with atypical clinical presentations, often mimicking stroke. The most common manifestations include neck pain, interscapular pain, radicular pain, paraplegia, and hemiplegia, which can even lead to long-term disability. These clinical presentations are similar to those of acute cerebral infarction, making misdiagnosis and missed diagnosis likely. The treatment of SSEH is primarily surgical, but in some cases, conservative medical treatment can be applied. We report a rare case of cervical SSEH where the patient’s condition improved rapidly with conservative medical therapy.

## 2 Clinical data

A 68 years-old male patient presented to our emergency department at 01:10:53 on 25 July 2022. Chief complaint: Neck pain accompanied by weakness and numbness of the left limbs for over 2 h. History: The patient and his family reported that around 23:00 last night, without any obvious cause, the patient experienced neck pain while resting, accompanied by weakness and numbness in the left limb. There were no symptoms of dizziness, headache, convulsions, altered consciousness, vomiting, diarrhea, chest tightness, chest pain, or incontinence of urine and stool. The patient denied any neck trauma and had not taken any medication on their own. The symptoms persisted without relief, prompting the patient to visit the emergency department of our hospital on their own. Past medical history: The patient has a history of nasopharyngeal carcinoma for many years, which has been cured; the specific diagnosis and treatment details are unknown. Bilateral hearing loss for over 20 years, the etiology is unknown, and the diagnosis and treatment details are unknown. The patient has a history of hypertension, the specific etiology of which remains unclear. The patient has not been on any antihypertensive medications and has not been monitoring blood pressure regularly. The patient denies a history of coronary heart disease and diabetes, and denies the use of antiplatelet or anticoagulant medications. Physical examination: Conscious, BP210/120 mmHg, no obvious abnormalities in heart and lung auscultation, abdomen flat and soft, no significant tenderness, refusal to have the back of the neck examined, muscle strength of the left limbs at grade 4, right limbs at grade 5, slightly deviated tongue extension to the right, symmetrical nasolabial folds, positive Babinski’s sign on the left side, sensory symmetry in both sides, symmetrical tendon reflexes present, and negative pathological signs on both sides. The electrocardiogram (ECG) examination indicated a sinus rhythm.

The preliminary diagnosis was an acute stroke, with the patient within the 4.5 h emergency thrombolysis window. An immediate non-contrast head CT scan was ordered, and the neurology department was urgently consulted.

**FIGURE 1:**
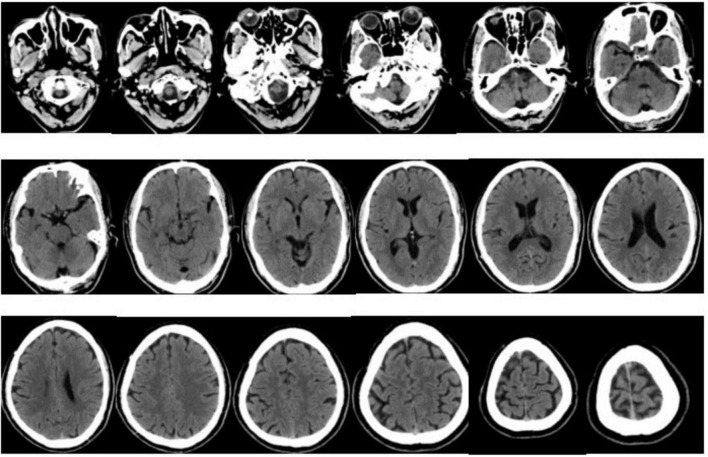
Bilateral cerebral hemispheres, cerebellum, and diencephalon were symmetrical, with clear boundaries between gray and white matter, and no definite abnormal density shadow was observed in the brain parenchyma definite abnormality was found in the skull bone. The C4–5 segments of bilateral internal carotid arteries were hyperdense. The paranasal sinus mucosa was slightly thickened.

At 01:28, A reading of the CT film (Figure 1) showed no obvious signs of acute cerebral hemorrhage. This is a sign that triggers the emergency thrombolysis for acute cerebral infarction.

**FIGURE 2:**
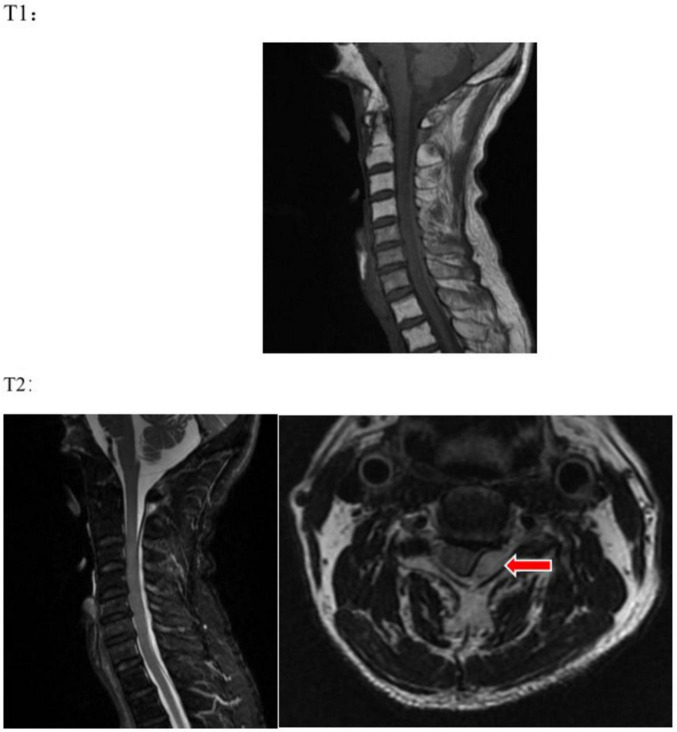
On T1 and T2 sequences, an abnormal signal is visible in the epidural space at the posterior part of the spinal canal from C2 to T1, which is suggestive of an epidural hemorrhage, with the spinal cord being compressed and displaced anteriorly and to the right. The red arrow indicates the site of the lesion.

However, the patient exhibited symptoms of neck pain, which do not entirely correspond to the symptoms of acute cerebral infarction. The patient has a history of hypertension, and it is crucial to differentiate vertebral artery dissection, as it is a contraindication for thrombolysis. At 01:39, urapidil was initiated to control the patient’s blood pressure. At 02:11, a complete neck vascular ultrasound showed no signs of dissection in the bilateral carotid and vertebral arteries. In the next step, it is necessary to differentiate between intervertebral disk protrusion and other acute cervical spine lesions. Since the onset of symptoms, 3 h and 10 min have passed, and the patient is still within the 4.5 h emergency thrombolysis window. We completed a magnetic resonance imaging (MRI) of the brain with diffusion-weighted imaging (DWI) to further ascertain the presence of acute cerebral infarction. Concurrently, a cervical MR scan was performed to assess whether intervertebral disk protrusion or other spinal canal lesions could be responsible for unilateral limb weakness. The MRI was completed at 02:44. The brain DWI sequence showed no significant radiological signs of acute cerebral infarction, thus ruling out acute cerebral infarction. The cervical MR scan (Figure 2) revealed: epidural hemorrhage at the posterior aspect of the spinal canal from the level of C2 to T1, with the spinal cord being compressed and displaced to the right anterior direction. This radiological finding correlates with the patient’s current clinical presentation. The diagnosis is now clear: cervical subdural epidural hematoma (SSEH).

Why does the patient have SSEH? Is there any abnormality in coagulation function? From 1:29 to 1:44, the emergency blood test results were reported sequentially. The complete blood count indicated that the white blood cells, red blood cells, and platelet counts were not significantly abnormal. Coagulation function tests showed that the Prothrombin Time (PT), Activated Partial Thromboplastin Time (APTT), and D-Dimer levels were all within the reference range. Electrolytes, bilirubin, and creatinine levels were also within the normal range. The main blood test indicators during the hospitalization treatment are presented in Table 1.

**TABLE 1:** Partial blood test indicators of the patient during hospitalization.

	2022-7-25	2022-7-26	2022-7-27	2022-7-28	2022-7-29	2022-7-30	2022-7-31	2022-8-1	2022-8-2
White blood cells (×10^9^/L)	6.66	7.76	5.51	6.16	5.50	6.9	8.04	–	–
Red blood cells (×10^12^/L)	4.33	3.97	3.93	4.12	3.65	3.95	3.98	–	–
Hemoglobin (g/L)	135	122	121	127	112	123	122	–	–
Platelets (×10^9^/L)	188	152	139	165	185	212	253	–	–
Sodium (mmol/L)	139.19	–	–	–	–	–	137.5	–	–
Potassium (mmol/L)	3.53	–	–	–	–	–	4.08	–	–
Potassium (mmol/L)	2.47	–	–	–	–	–	2.21	–	–
Total Bilirubin (umol/L)	10.15	–	29.4	13.01	14.66	8.59	–	–	–
Albumin (g/L)	46.97	–	34.55	33.15	34.24	32.76	–	–	–
Creatinine (umol/L)	76.76	–	56.56	–	–	–	57.12	–	–
PT (秒)	11.6	13.4	14.9	–	–	12.6	–	–	–
APTT (秒)	25.6	31.9	32.4	–	–	29.9	–	–	–
D-Dimer (mg/L)	<0.2	0.8	0.8	–	–	–	–	–	–

We invited urgent consultations with neurologists and neurosurgeons to formulate an initial treatment plan. The patient has a lesion in the high cervical segment with a risk of sudden respiratory arrest, necessitating transfer to the ICU for monitoring of vital signs. For pharmacological treatment, 125 ml of 20% mannitol injection is administered twice daily (bid), and urapidil or nicardipine is used intravenously to control blood pressure at 90–100/60–90 mmHg. The patient’s current spinal cord injury is classified as Frankel Grade D, with no trend of worsening spinal cord neurological symptoms, thus conservative treatment can be temporarily adopted with active blood pressure control. If there is an aggravation of spinal cord neurological injury symptoms, timely surgical intervention can be considered. Given the patient’s neck pain and spinal cord compression, a cervical collar can be used to immobilize the neck, reducing neck movement.

**FIGURE 3:**
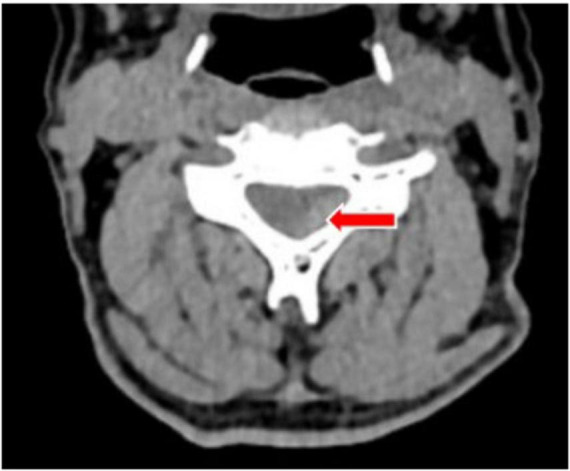
A plain computed tomography (CT) scan showed a strip of high-density shadow in the posterior part of the cervical spinal canal, with a CT value of about 71 HU, and the spinal cord was slightly compressed and displaced in the right front. The site of the lesion is indicated by the red arrow.

At 04:00, a cervical collar was applied to immobilize the patient’s neck, and the patient was transferred to the ICU for continued treatment. At 05:27, a CT plain scan + CTA (Computed Tomography Angiography) of the head and neck was completed. The CT plain scan images suggested a strip-like high-density shadow at the posterior part of the spinal canal from C2 to C7 levels, with a CT value of approximately 71 HU, indicating slight compression and right anterior displacement of the spinal cord, suggesting the possibility of epidural hemorrhage (Figure 3). The CTA did not reveal any obvious arteriovenous malformations.

**FIGURE 4:**
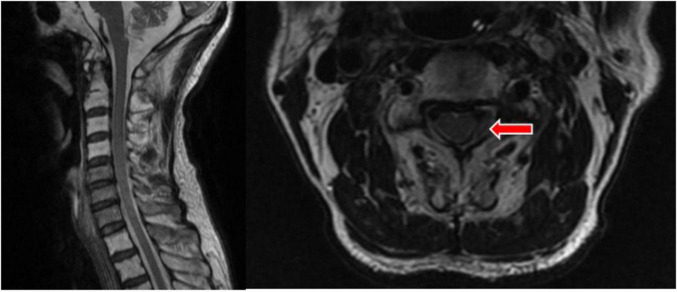
T2 showed a left epidural hematoma at the level of the C3–4 vertebral body, with reduced range and thickness of the lesion. The site of the lesion is indicated by the red arrow.

On 26 July 2022, at 16:26, a follow-up cervical MRI was performed. It revealed an epidural hematoma on the left side at the level of the C3–C4 vertebral bodies, with compression of the adjacent cervical spinal cord and the left nerve root and a reduction in the extent and thickness of the lesion compared to the previous one (Figure 4). The patient’s neck pain has essentially disappeared, and the muscle strength on the left side has recovered to grade 5.

In the subsequent treatment and diagnosis, the clinical manifestation of the patient’s neck pain rapidly improved within 24 h. Follow-up imaging showed a significant reduction in the size of the hematoma. Consultations from neurosurgery and spinal surgery both agreed to continue with conservative treatment. On 28 July 2022, intravenous antihypertensive therapy achieved the target blood pressure, and amlodipine tablets were added orally. The intravenous antihypertensive medication was gradually tapered according to the target blood pressure until it was discontinued. On 1 August 2022, the patient’s muscle strength in the left limb had essentially returned to normal. After consultation with spinal surgery, it was decided to discontinue mannitol. On 2 August 2022, the patient reported the disappearance of neck pain. At discharge, the patient’s muscle strength in the left limb was grade 5, capable of self-care, and satisfied with the treatment outcome. The patient was discharged with a cervical collar and antihypertensive medication, with instructions for outpatient follow-up.

## 3 Discussion

Spontaneous spinal epidural hematoma (SSEH) is a rare condition, referring to spontaneous bleeding in the spinal epidural space without surgery or trauma. It was first reported by Jackson R. in 1869, with an incidence rate of approximately 0.1/100,000 people (1). The age of onset for SSEH is typically between 40 and 60 years, with a male-to-female incidence ratio of 1.4:1 (2). The classic clinical presentation of SSEH includes sudden back pain, followed by sensory and motor deficits and bowel and bladder dysfunction below the lesion level caused by compression of the nerve roots or spinal cord by the hematoma. There may be a delay from the onset of back pain to the appearance of neurological manifestations, with symptom onset ranging from a few hours to several days or even months (3). According to existing research, the most common risk factors that can lead to SSEH include anticoagulant therapy, hypertension, antiplatelet therapy, anemia, diabetes, hypothyroidism, pregnancy, and deep diving (4).

The pathogenesis of SSEH remains unclear. Currently, there are two prevailing theories to explain this condition. The “venous theory” suggests that the vertebral venous system is a low-pressure system without venous valves and is connected to the thoracoabdominal venous system. When there is an increase in intrathoracic or intra-abdominal pressure, the pressure within the vertebral venous plexus increases, making the dorsal epidural venous plexus prone to rupture, leading to SSEH (5). The “arterial theory” posits that, for patients with hypertension or those using anticoagulants, the epidural arteries are more susceptible to irritation and thus more likely to rupture, causing the disease (6). If the progression of neurological compression symptoms is rapid, it may be due to arterial rupture; otherwise, it is likely due to venous rupture (7, 8).

Stroke mimics refer to non-vasculogenic diseases in clinical practice that present with clinical manifestations similar to stroke, which can manifest as acute hemiplegia of the limbs and partially overlap with the clinical presentation of Spontaneous Spinal Epidural Hematoma (SSEH). Acute cerebral ischemic stroke has a high incidence rate and a high risk of causing disability, with typical clinical manifestations such as hemiplegia and aphasia, and the main treatment plan is thrombolysis. For patients inadvertently treated with thrombolysis for stroke mimics, once the diagnosis is confirmed, the use of thrombolytic drugs should be immediately discontinued. If the medication has already been administered, the patient’s condition should be closely monitored for at least 24 h (9).

Spontaneous spinal epidural hematoma cannot be detected on routine head CT scans and is easily subjected to the initiation of the stroke thrombolysis process. In recent years, there have still been case reports of SSEH being misdiagnosed as ischemic stroke and treated with thrombolysis. As a hemorrhagic disease, thrombolysis can lead to rapid exacerbation of symptoms. Studies have found that almost all patients with spinal epidural hematomas experience pain, including headache, neck pain, and back pain (10, 11). The sensitivity of pain as a predictive factor for spinal epidural hematoma is 100%, the specificity is 88.7%, the positive predictive value is 2.3%, and the negative predictive value is 100%, which may be a potential predictive factor for the presence of SSEH. For some patients with mild SSEH, neurological deficits often resolve spontaneously, hence, some patients may also be misdiagnosed as having a transient ischemic attack (12).

In the imaging examination of SSEH, MRI is currently considered the best auxiliary examination for SSEH (13), and the signal evolution of the hematoma on MRI plain scans at different time periods shows certain regularity. Within 24 h, it can present as isointense on T1 and hyperintense on T2, between 24 and 48 h it can be hyperintense on both T1 and T2, between 48 and 240 h it can show heterogeneous enhancement on T1, and after 240 h it can be hyperintense on both T1 and T2 (3, 14). CT examination has the advantages of being rapid, convenient, and economical. In Chinese literature, it is reported that the imaging of SSEH often shows an epidural hematoma in the posterior-lateral aspect of the spinal canal, replaced by the hematoma, presenting as a high-density soft tissue shadow with CT values between 50 and 90 HU, with clear boundaries, the posterior edge of the corresponding segmental dural sac being compressed and deformed, and the spinal cord may show varying degrees of compression. It can differentiate between acute hemorrhage, fat, and bony structures in the spinal canal, with the hematoma appearing as a spindle or strip shape on the sagittal view, and arc-shaped or crescent-shaped on the transverse section. However, CT is not sensitive in distinguishing during the subacute phase.

Currently, there are no guidelines for the treatment strategy of SSEH. The main goal of treating SSEH is to quickly relieve spinal cord compression, restore neurological function, and prevent complications. The treatment plan should be formulated based on the specific situation of the patient, including surgical treatment and conservative treatment. The indications for conservative treatment in SSEH patients are: the presence of surgical contraindications, a Frankel grade of E, or a Frankel grade of C/D with a trend of gradual recovery of neurological function. The selected pharmacological treatment regimen may comprise corticosteroids, mannitol, (if necessary) neurotrophic agents, analgesia as required, and symptomatic supportive therapy, among others. During the treatment process, bed rest is mandatory to avoid large-scale movements and vigorous coughing that can increase thoracic and abdominal pressure. Concurrently, close monitoring of the patient’s neurological signs is essential. The selected pharmacological treatment regimen may comprise corticosteroids, mannitol, (if necessary) nutritional neurology, on-demand analgesia, symptomatic supportive treatment, etc. During the treatment process, bed rest is required to avoid actions that increase thoracoabdominal pressure such as large movements, severe coughing, etc., and to closely observe the patient’s neurological signs. For SSEH patients with a Frankel grade of A, B, or C, adopting a conservative treatment strategy will often prolong the hospital stay, and the recovery process of neurological function is usually slow, sometimes even further deterioration of neurological dysfunction may occur, which may lead to permanent spinal cord neurological damage. Therefore, it is recommended that these patients should receive surgical treatment as early as possible (15). Choosing the best timing for surgery is crucial, and the common view is that surgery within 24 h after the onset of the disease can achieve the best results. However, for patients with urgent conditions or rapidly deteriorating symptoms, surgery can be performed immediately within a few hours after the onset of the disease. The surgical method chosen is laminectomy and hematoma removal, the main purpose is to remove the hematoma, alleviate the compression of the spinal cord, and restore the normal blood supply and neurological function of the spinal cord. Postoperatively, the treatment regimen continued with mannitol and corticosteroids, among other medications. This approach is beneficial for effectively alleviating spinal cord edema and accelerating postoperative neurological recovery. Concurrently, it is crucial to monitor vital signs and closely observe the patient’s neurological signs. Any abnormalities should be promptly addressed.

The prognosis of acute spinal epidural hematoma is related to various factors, such as the size and location of the hematoma, the severity of symptoms, the timing of surgery, and postoperative care. Generally speaking, patients with early diagnosis, timely treatment, and proper postoperative care have a better prognosis, but some patients may still suffer from varying degrees of neurological dysfunction.

The SSEH patient reported in this case study had no obvious precipitating factors before the onset of the disease and denied any history of trauma; the patient had a past medical history of hypertension but did not adhere to a regular medication regimen and did not monitor their blood pressure. At the time of presentation, the patient was in a state of Grade 3 hypertension, which is a risk factor for both cerebral infarction and SSEH. A non-contrast head CT scan showed no significant hemorrhagic foci, the time since onset was less than 4.5 h, and there was no recent history of bleeding disorders, all of which are criteria for triggering the stroke thrombolysis green channel. However, the patient experienced persistent neck pain that occurred concurrently with unilateral limb weakness, which did not match the clinical presentation of a stroke, necessitating differentiation from conditions such as carotid artery dissection and acute spinal cord lesions. We promptly performed carotid and vertebral artery ultrasounds, which showed no signs of arterial dissection. Further magnetic resonance imaging (MRI) with diffusion-weighted imaging (DWI) sequence revealed no significant acute infarction foci. A cervical spine MRI showed an abnormal density shadow within the spinal canal, and in conjunction with the clinical presentation, a definitive diagnosis of high cervical segment SSEH was established. This condition carries a risk of sudden respiratory and cardiac arrest, and aggressive surgical intervention should be highly considered. However, the patient’s Frankel grade was D, and a computed tomography angiography (CTA) showed no significant arteriovenous malformations. After aggressive blood pressure control and cervical immobilization with a cervical collar, the patient was closely monitored in the ICU without worsening the condition and showed improvement within 24 h. A follow-up cervical MRI showed a significant reduction in the hemorrhagic lesion, thus surgery could be temporarily avoided. After a week of close observation and treatment, the patient’s muscle strength returned to normal, and blood pressure was maintained within an acceptable range, indicating that conservative treatment was effective.

The patient arrived at the emergency department within 2 h of the onset of symptoms, allowing ample time for thorough examinations. The attending and consulting physicians astutely detected potential stroke-like signals and did not blindly initiate thrombolysis. A definitive diagnosis of SSEH was established within 3 h and 45 min of symptom onset, thereby averting severe consequences that could have resulted from misdiagnosis and inappropriate thrombolysis.

## 4 Conclusion

Spontaneous spinal epidural hematoma (SSEH), although rare in clinical practice, is not difficult to diagnose. The main diagnostic challenge may lie in the fact that many physicians lack clinical experience with this rare condition, and some may even misdiagnose such patients as having acute cerebral infarction and initiate emergency thrombolysis. The clinical manifestations of SSEH are relatively typical, with spinal pain and neurological dysfunction at the corresponding spinal cord level being almost universally present. Most patients have a history of hypertension or use of anticoagulants. Given that many hospitals do not have the capability for emergency MRI, an emergency non-contrast neck CT scan also has diagnostic value for this condition. Combined with clinical manifestations, it can effectively differentiate SSEH from acute stroke. In the conservative treatment protocol for cervical SSEH, we innovatively propose that the use of a cervical collar may be an effective adjunctive therapeutic approach.

## Data Availability

The original contributions presented in this study are included in this article/supplementary material, further inquiries can be directed to the corresponding author.
